# Audit of Renal Function Monitoring and Indications in Patients Prescribed Memantine at Ribchester Centre: A Review of Compliance With Pre-Prescription Guidelines

**DOI:** 10.1192/bjo.2025.10618

**Published:** 2025-06-20

**Authors:** Laiba Khalid, Akash Kumar

**Affiliations:** Pennine Care NHS Trust, Manchester, United Kingdom

## Abstract

**Aims:** To assess whether renal function tests are appropriately reviewed prior to the prescription of memantine, in accordance with NICE clinical guidelines, ensuring the safety and effectiveness of medication.

To assess if memantine is prescribed according to the indications listed in NICE guidelines.

**Methods:** This retrospective audit reviewed patients with a diagnosis of dementia, seen at old age psychiatry outpatient clinic Bury from 1 August to 31 October 2024.

The review focused on patients starting memantine during this period, assessing whether renal function was evaluated prior to initiation, the indication for memantine use, and whether it was prescribed as monotherapy or adjunctive therapy.

The targeted population included patients over 18 years, with a sample size of 395 (August: 118, September: 151, October: 126). Data were collected by reviewers from 1 to 15 November, using letters on Paris and online care records, and recorded in an Excel sheet.

**Results:** Of the 12 patients recommended for memantine, only three had a diagnostic indication explicitly documented in their letters, in alignment with NICE guidelines.

In two cases, memantine was prescribed as an adjunct therapy alongside acetylcholinesterase inhibitors – donepezil in one instance and rivastigmine in the other.

For one patient, memantine was utilized specifically for the management of severe Alzheimer’s disease.

All patients had their estimated glomerular filtration rate (eGFR) and renal function test (RFT) documented in the GM records.

For seven patients, these details were not included in their correspondence but present on GM record.

For the remaining five patients the letters either explicitly mentioned the eGFR or referenced the eGFR.

**Conclusion:** Guideline adherence: Only 3 out of 12 memantine prescriptions had diagnostic indications documented per NICE guidelines, indicating incomplete compliance.

Prescribing practices: Memantine was used appropriately as adjunct therapy in two cases and for severe Alzheimer’s in one case.

Renal function monitoring: While all patients had eGFR and renal function tests documented in GM records, these details were not clearly recorded in correspondence for 7 patients, highlighting communication gaps.

